# Use of manual and powered wheelchair in children with cerebral palsy: a cross-sectional study

**DOI:** 10.1186/1471-2431-10-59

**Published:** 2010-08-16

**Authors:** Elisabet Rodby-Bousquet, Gunnar Hägglund

**Affiliations:** 1 Department of Orthopaedics, Lund University, University Hospital, 221 85 Lund, Sweden; 2 Centre for Clinical Research, Uppsala University, Central Hospital, 721 89 Västerås, Sweden

## Abstract

**Background:**

Mobility is important for the cognitive and psychosocial development of children. Almost one third of children with cerebral palsy (CP) are non-ambulant. Wheelchairs can provide independent mobility, allowing them to explore their environment. Independent mobility is vital for activity and participation and reduces the dependence on caregivers. The purpose of this study was to describe the use of manual and powered wheelchair indoors and outdoors in relation to the degree of independent wheelchair mobility or need for assistance in a total population of children with CP.

**Methods:**

A cross-sectional study was performed including all children aged 3-18 years with CP living in southern Sweden during 2008. Data was extracted from a register and health care programme for children with CP (CPUP). There were a total of 562 children (326 boys, 236 girls) in the register. Information on the child's use of manual and powered wheelchair indoors and outdoors and the performance in self-propelling or need for assistance were analysed related to age, CP subtype and gross motor function.

**Results:**

Wheelchairs for mobility indoors were used by 165 (29%) of the 562 children; 61 used wheelchair for independent mobility (32 using manual only, 12 powered only, 17 both) and 104 were pushed by an adult. For outdoor mobility wheelchairs were used by 228 children (41%); 66 used a wheelchair for independent mobility (18 using manual only, 36 powered only, 12 both) and 162 were pushed. The use of wheelchair increased with age and was most frequent in the spastic bilateral and dyskinetic subtypes. Most powered wheelchairs were operated by children at GMFCS level IV.

**Conclusion:**

In this total population of children with CP, aged 3-18 years, 29% used a wheelchair indoors and 41% outdoors. A majority using manual wheelchairs needed adult assistance (86%) while powered wheelchairs provided independent mobility in most cases (86%). To achieve a high level of independent mobility, both manual and powered wheelchairs should be considered at an early age for children with impaired walking ability.

## Background

Cerebral palsy (CP) has been defined as a group of permanent disorders of the development of movement and posture, causing activity limitation, that are attributed to non-progressive disturbances that occurred in the developing fetal or infant brain [1]. The severity of impairments varies greatly and the children's mobility ranges from independent walking to totally dependent wheelchair mobility and almost one third is non-ambulant [2].

Mobility is important for the cognitive and psychosocial development of children [3,4]. Independent mobility is vital for activity and participation, reducing dependence on caregivers and the environment [3-5]. The single most important factor for the experience of participation in adolescents with disabilities is the possibility to be "where it happens", which is closely related to independent mobility [6]. Independent mobility is also important for self-sufficiency [6].

Mobility is influenced by the environment. According to the International Classification of Functioning, Disability and Health (ICF) [10] the environment is defined as the physical, social and attitudinal conditions that are present in an individual's life. Assistive products and technology are defined as any product, instrument, equipment or technology adapted or specially designed for improving the functioning of a disabled person. Assistive devices such as wheelchairs can provide independent mobility to children with disabilities, allowing them to explore their environment [3-5,7]. Mobility devices improve activity, participation, user satisfaction and quality of life [8].

Performance, what a child "does do", can differ from capability, what a child "can do" [9,10]. Environmental factors, influence of parents, personal factors (e.g. choice, motivation, acceptance of disability) and body functions (e.g. fatigue) are important when it comes to use a manually propelled wheelchair, powered wheelchair or none, both indoors and outdoors. Knowledge of the degree of independent mobility and the use of manual and powered wheelchair in a total population of children with CP may be useful for health care planning and for facilitating early independent mobility for the individual child.

A register and health care programme for children with CP (CPUP) was initiated in southern Sweden in 1994 [11,12]. Data from this register has been used to analyse the use of wheelchair and the degree of independent wheelchair mobility in children with cerebral palsy.

### Purpose

To describe the use of manual and powered wheelchair indoors and outdoors in relation to the degree of independent wheelchair mobility or need for assistance for different levels of gross motor function, CP subtypes and age groups in a total population of children with CP.

## Methods

The CPUP register includes all children with CP born after 1 January 1990 living in the counties of Skåne and Blekinge in southern Sweden, which have a total population of about 1.3 million. The number of children with CP in the area corresponds to a prevalence of 2.4 per 1000 live births [13,14]. The programme includes a continuous standardized follow-up of gross and fine motor function, clinical findings and treatment. The child is examined by its local physiotherapist annually. The assessment form includes information on the child's use of manual and powered wheelchair for mobility indoors and outdoors and whether the child self-propels or need adult assistance.

To obtain information on the child's wheelchair performance, the children and their caregivers answered the following questions: Does the child usually use a: (1) Manual wheelchair for mobility indoors? (2) Powered wheelchair for mobility indoors? (3) Manual wheelchair for mobility outdoors? (4) Powered wheelchair for mobility outdoors? The options were: (A) No, the child does not use a wheelchair; (B) Yes, the child self-propels/operates independently; (C) Yes, the child is pushed by an adult.

The results were analysed related to CP subtype, gross motor function and age. The CP subtypes were classified as Spastic unilateral, Spastic bilateral, Dyskinetic, Ataxic and Unclassified (or mixed), according to the Surveillance of Cerebral Palsy in Europe network (SCPE) [15]. The gross motor function was classified according to the Gross Motor Function Classification System (GMFCS) [16,17] which is an age-related five-level system based on functional limitations, in which level I is the most independent and level V is the least independent. The CP subtype for each child was determined by the child's neuropaediatrician and the GMFCS level by its local physiotherapist. To analyse differences in data at different ages the children were divided into different age groups according to the Swedish school system: 3-6, 7-9, 10-12, 13-15 and 16-18 years.

A cross-sectional study was performed based on data from the CPUP register including all children aged 3-18 years with CP living in the southern parts of Sweden during 2008. There were in total 562 children in the register, 326 boys and 236 girls born 1990-2005. The distribution of age, sex, GMFCS level and CP subtype of the 562 children is presented in Table 1. There were no significant differences in GMFCS levels or classified CP subtypes between the age groups. However, in the pre-school children, aged 3-6 years, more children were unclassified (UC).

**Table 1:** Details of the 562 children

Age	No of Children	Sex		GMFCS level					CP subtype				
		Boys	Girls	I	II	III	IV	V	S U	S B	DY	AT	UC
**3-6**	116	67	49	51	9	21	16	19	25	27	21	7	36
**7-9**	104	53	51	52	14	8	19	11	35	38	13	9	9
**10-12**	117	67	50	51	22	14	17	13	38	47	17	5	10
**13-15**	117	78	39	59	14	7	18	19	37	49	16	13	2
**16-18**	108	61	47	51	17	14	14	12	28	48	16	14	2

**Total**	562	326	236	264	76	64	84	74	163	209	83	48	59

SU = Spastic unilateral CP, SB = Spastic bilateral CP, DY = Dyskinetic CP, AT = Ataxic CP, UC = Unclassified CP

SPSS version 17.0 was used for the statistical analyses. Linear by linear association test was used for analysing trends in wheelchair use related to GMFCS levels and to age. Kruskal Wallis test was used for analysing wheelchair use related to CP subtypes. Spearman's rank correlation test was used to calculate correlations for ordinal data. Pearson Chi square test was used to analyse categorical data. P-values < 0.05 were considered significant.

The study was approved by the Medical Research Ethics Committee at Lund University (LU-443-99).

## Results

### Indoor mobility

Wheelchairs for mobility indoors were used by 165 children (29%). Information was missing in 6 of the 562 children. Of the 165 children, 61 used a wheelchair for independent mobility (32 using manual only, 12 powered only, 17 both) and 104 were pushed by an adult (Table 2). Manual wheelchairs were used by 163 children, of which 49 (30%) self-propelled and 114 (70%) were pushed. Powered wheelchairs were used by 35 children, of whom 29 (83%) operated independently while 6 (17%) used powered wheelchairs operated by an adult (Table 2).

**Table 2:** Number of children using manual and powered wheelchair indoors

		Powered			
		Do not use	SM	AA	Total
**Manual**	**Do not use**	391	2	0	393
	**SM**	32	17	0	49
	**AA**	98	10	6	114
	**Total**	521	29	6	556

SM = self-mobility, AA = Adult assistance. Information missing in 6 of the 562 children.

**Figure 1:**
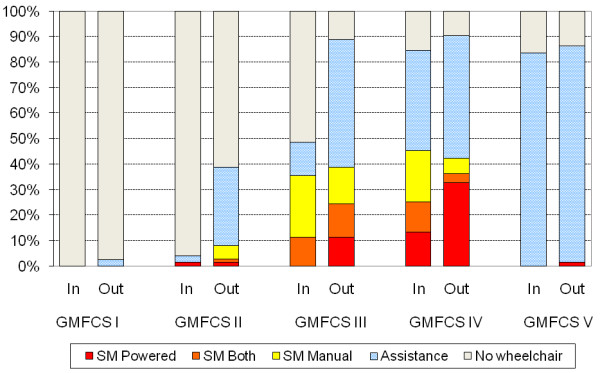
**Number of children (%) in different GMFCS levels using wheelchair for self-mobility (SM) and for assisted mobility indoors and outdoors**. SM Powered = operates a powered wheelchair; SM Both = self-propels both manual and powered wheelchairs; SM Manual = self-propels a manual wheelchair; Assistance = use manual or powered wheelchairs but do not self-propel either of them and need adult assistance. Linear by linear association test showed an increase in the use of manual and powered wheelchair indoors with GMFCS level (p < 0.001).

The use of manual and powered wheelchair indoors increased with GMFCS level (p < 0.001) (Figure 1). At GMFCS level II 4% used a manual or powered wheelchair indoors, 48% at level III and 84% at levels IV-V. A majority (73%) of the wheelchair users at level III were independent (using manual and/or powered wheelchairs). Powered wheelchairs were most frequent at GMFCS level IV. Of all the children at level IV 45% manoeuvred manual or powered wheelchairs independently while 39% were pushed by an adult. At level V all wheelchair users had manual wheelchairs and required assistance (Figure 1).

**Figure 2:**
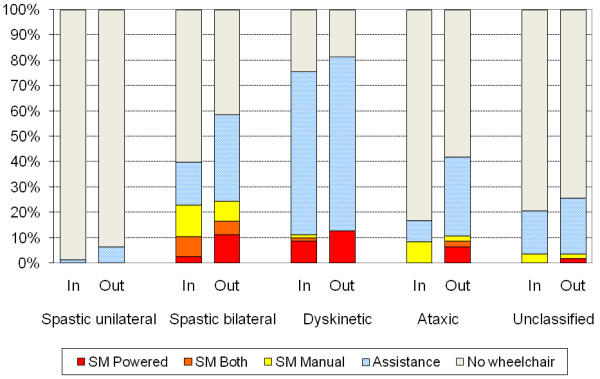
**Number of children (%) in different CP subtypes using wheelchair for self-mobility (SM) and for assisted mobility indoors and outdoors**. SM Powered = operates a powered wheelchair; SM Both = self-propels both manual and powered wheelchairs; SM Manual = self-propels a manual wheelchair; Assistance = use manual or powered wheelchairs but do not self-propel either of them and need adult assistance. Kruskal Wallis test showed a significant difference in wheelchair use between the subtypes (p < 0.001).

There was a difference in use of wheelchair between CP subtypes (p < 0.001) (Figure 2). The use of wheelchair for indoor mobility was most frequent within the dyskinetic subtype (76%), of those 11% self-propelled (1% using manual wheelchair, 9% powered, 1% both). All children operating powered wheelchairs indoors had spastic bilateral or dyskinetic CP. In the spastic bilateral subtype 40% used wheelchairs, of those 23% self-propelled (manual and powered) and 17% were pushed in manual wheelchairs. Of the children with ataxic CP 16% used manual wheelchairs, of those half self-propelled and the other half were pushed (Figure 2).

The use of manual wheelchairs indoors increased with age (p = 0.001) but the change was not significant for powered wheelchairs (Table 3). The youngest child who had independent wheeled mobility indoors was 3 years old and used a manual wheelchair. For distribution of manual and powered wheelchairs and independent mobility in different age groups, see Table 3. The use of wheelchairs for indoor mobility was similar for boys and girls (manual wheelchair: boys 30%, girls 29%, powered wheelchair: boys 7%, girls 5%).

**Table 3:** Use of manual and powered wheelchair indoors and outdoors (%) related to age

Environment	Wheelchair	Age (years)	Self-propels %	Adult assistance %	Do not use %
**Indoors**	**Manual**	3-6	5.2	10.4	84.3
		7-9	8.7	25.2	66.0
		10-12	9.5	22.4	68.1
		13-15	11.1	25.6	63.2
		16-18	10.2	18.5	71.3
	
	**Powered**	3-6	4.3	0	95.7
		7-9	1.9	1.0	97.1
		10-12	6.1	0	93.9
		13-15	7.7	2.6	89.7
		16-18	5.7	1.9	92.5
	
**Outdoors**	**Manual**	3-6	3.5	24.3	72.2
		7-9	1.9	43.7	54.4
		10-12	8.7	39.1	52.2
		13-15	6.8	33.3	59.8
		16-18	7.5	32.7	59.8
	
	**Powered**	3-6	4.3	0	95.7
		7-9	5.9	2.0	92.2
		10-12	8.0	0.9	91.2
		13-15	13.7	2.6	83.8
		16-18	11.4	1.9	86.7

Linear by linear association test showed an increase in use of manual wheelchairs indoors (p = 0.001) and outdoors (p = 0.003) with age. The use of powered wheelchairs outdoors increased with age (p = 0.033) but the change was not significant for powered mobility indoors.

### Outdoor mobility

Wheelchairs for outdoor mobility were used by 228 children (41%). Information was missing in 10 of the 562 children. Of the 228 children, 66 used a wheelchair for independent mobility (Table 4). For outdoor mobility 219 children used manual wheelchairs, 30 (14%) self-propelled and 189 children (86%) were pushed. Powered wheelchairs were used by 56 children, of whom 48 (86%) operated independently and 8 (14%) used powered wheelchairs operated by an adult (Table 4).

**Table 4:** Number of children using manual and powered wheelchair outdoors

		Powered			
		Do not use	SM	AA	Total
**Manual**	**Do not use**	324	6	3	333
	**SM**	18	12	0	30
	**AA**	154	30	5	189
	**Total**	496	48	8	552

SM = self-mobility, AA = adult assistance. Information missing in 10 of the 562 children.

Wheelchair for outdoor mobility was more frequent than indoors and the use of both manual and powered wheelchair outdoors increased with GMFCS levels (p < 0.001) (Figure 1). At GMFCS level I 2% used a wheelchair, 39% at level II and 85-90% at levels III-V. All the 25 children at GMFCS levels III-V who did not use a wheelchair for outdoor mobility were aged 3-6 years. Only one child at GMFCS level V operated a powered wheelchair outdoors. More children at GMFCS level III and IV used powered wheelchairs for independent mobility outdoors than indoors. There was also an increase in the number of children needing adult assistance at all GMFCS levels (Figure 1).

There was a significant difference in wheelchair use between the subtypes (p < 0.001) (Figure 2). The use of wheelchair for outdoor mobility was most frequent within the dyskinetic subtype, where 69% were pushed in manual wheelchairs and 13% operated powered wheelchairs independently. More children with spastic bilateral CP had independent wheeled mobility compared to the other subtypes, in total 24% (11% using powered wheelchairs, 8% manual and 5% both). Of the children with ataxic CP 42% used wheelchairs outdoors, of those 11% were independent and 31% were pushed (Figure 2).

The use of both manual wheelchairs (p = 0.003) and powered wheelchairs (p = 0.033) increased with age (Table 3). No child less than 4 years old had independent wheeled mobility outdoors. The use of wheelchairs for outdoor mobility was similar for boys and girls (manual wheelchair: boys 40%, girls 41%, powered wheelchair: boys 11%, girls 9%).

There was a correlation between the use of manual wheelchairs indoors and outdoors (r_s _= 0.722) and between powered wheelchair indoors and outdoors (r_s _= 0.628). There was also a correlation between GMFCS level and use of manual wheelchairs indoors (r_s _= 0.726) and outdoors (r_s _= 0.719) but not for the use of powered wheelchairs.

## Discussion

This is, to our knowledge, the first study demonstrating the use of wheelchair in a total population of children with CP, showing the degree of independent wheelchair mobility and the use of adult assistance for mobility. Almost all (98%) children with CP in the area and their families agree to participate in the CPUP programme [14].

The results reflect the children's performance, what they usually do, not their capability, what they can do [9,10]. A child's performance can relate to differences in the environmental and personal factors. In recent years more attention has been paid to altering the environment in order to compensate for functional impairment [7,18-20].

This study describes the performance of wheeled mobility in different environments (indoors and outdoors) and whether the children use a manual wheelchair, powered wheelchair or both. However it only shows their most common performance in each wheelchair and environment. The register does not provide detailed information on the type of wheelchair.

Manual wheelchairs for self-mobility were most frequent in children at GMFCS level III while powered wheelchairs were most frequent at level IV. Children at level III-IV achieved a higher degree of independent mobility using manual and powered wheelchairs. This corresponds to the results seen in the study by Östensjö et al. 2005 [18], where the largest increase in mobility by using wheelchair was seen at GMFCS level IV.

Postural instability restricts functional performance and upper extremity function in children with CP [21]. For outdoor mobility 86% using a manual wheelchair needed adult assistance and only 14% self-propelled. Lacoste et al. [21] found that children with CP who self-propelled their wheelchairs had difficulties in driving due to postural instability. Of the children using manual wheelchairs, 89% became unstable when propelling while 61% did when operating their powered wheelchairs. Powered mobility improves independence in mobility, and providing a stable sitting posture is essential to improve function and wheeled mobility.

Wheelchairs were most frequent in children with dyskinetic CP. Arner et al. 2008 [22] reported difficulties with manual activities in 80% of children with dyskinetic CP, in 41% of those with ataxia and in 39% of those with spastic bilateral CP. The reduced hand function may be one explanation to the fact that no child with dyskinetic CP self-propelled a manual wheelchair outdoors while 77% of those having a powered wheelchair did. Of those using manual wheelchairs, only 10% of the ataxic subtype and 25% of those with spastic bilateral CP, self-propelled while 92% of the children in these subtypes using powered wheelchairs operated independently. The result indicates that most children with dyskinetic CP need a powered wheelchair to achieve independent wheeled mobility. Some children with ataxic or spastic bilateral CP may be able to self-propel a manual wheelchair but most of them are more likely to become independent using a powered wheelchair. However, powered wheelchairs require more training and more space to operate and are not as easily transported so environmental and personal factors must be considered.

Palisano et al. [5] analysed the mobility methods in Ontario, Canada. Among 360 children aged 4-12 years of age at GMFCS level III-V, 67% were pushed in manual wheelchairs outdoors, 7% used a manual wheelchair for self-mobility and 12% operated a powered wheelchair. The corresponding figures for the same age groups in the present study were almost equal: 62% were pushed, 6% self-propelled in manual wheelchairs and 15% operated powered wheelchairs. The age when the children started to use powered wheelchairs cannot be compared. Manual and powered wheelchairs are provided free of charge by the Assistive Technology Centres in Sweden. Consequently, the results reflect the children's use of wheelchairs without regard to the families' economic situation. However, the results from the Canadian study show similar results in spite of different financial systems.

Of the total material (GMFCS I-V) only 5 children (4%) used a powered wheelchair before school age (7 years). Of the children aged 7-18, 13% used a powered wheelchair.

Only one child at GMFCS level V had independent wheelchair mobility outdoors using a powered wheelchair, even though early self-produced mobility is crucial for the child's cognitive and psychosocial development [3]. Bottos et al. [23] showed that 21 of 27 children with severe motor disability aged 3-8 years (mean 6 years 3 months) were able to operate a powered wheelchair with no or minimal adult assistance. A majority of the parents were opposed to the idea of a power wheelchair initially, but after provision almost all were positive. Butler [24] reported improved self-initiated behaviours such as interaction with objects, communication and changes in location in children aged 23-38 months provided with powered mobility. Children with motor impairments may be at risk of developing learned helplessness if their development of independence is not supported [19].

In spite of the benefits connected to independent mobility there is sometimes a resistance to prescribe a wheelchair to a young child. This might be due to a belief that the child's most normal motor skill must be used and the improvement of walking abilities might be prevented by the use of a wheelchair [7]. This philosophy may also consider powered wheelchairs as inappropriate for a child who has the ability to use a manual wheelchair. A wheelchair is often used as a symbol of handicap or disability, and assistive devices are designed to compensate for disability, so the stereotype of disability is easily reflected by them [25]. Acceptance of disability usually leads to a greater acceptance and use of devices [25].

Franks et al. 1991 [26] reported that mobility methods affect school performance; the use of a wheelchair had a less negative impact on visuomotor accuracy than walking with assistive devices due to a lower energy cost. Participation and social interaction opportunities in the school environment also improved with the use of assistive devices in children with CP [25].

Independent mobility improves with the use of powered wheelchairs, while manual wheelchairs mainly ease the care [18]. Our study supports those results since 86% of the children using powered wheelchairs operated independently, while 14% of the children using manual wheelchair self-propelled and a majority (86%) were pushed. The 25 children at GMFCS levels III-V who did not use a wheelchair for outdoor mobility were all aged 3-6 years and might be seated in a stroller/buggy outdoors.

## Conclusions

In this total population of children with CP 29% used a wheelchair indoors and 41% outdoors. A majority of the children using manual wheelchairs were pushed by an adult (86%) while powered wheelchairs provided independent mobility in most cases (86%). The results indicate that most children with dyskinetic CP need a powered wheelchair to achieve independent wheeled mobility. Some children with ataxia (10%) or spastic bilateral CP (25%) may be able to self-propel a manual wheelchair but most of them are more likely to become independent using a powered wheelchair. To achieve as high a level of independent mobility as possible, both manual and powered wheelchairs should be considered at an early age for children with impaired walking ability.

## Abbreviations

CP: Cerebral Palsy; CPUP: National Health Care Quality Register for Cerebral Palsy; GMFCS: Gross Motor Function Classification System; SCPE: Surveillance of Cerebral Palsy in Europe network

## Competing interests

The authors declare that they have no competing interests.

## Authors' contributions

ERB and GH designed the study. Both authors analysed the results. ERB wrote the first draft, which was then actively improved and revised by both authors. Both authors approved the final manuscript.

## Authors' information

ERB is a PT at the Department of Orthopaedics, Lund University, SE 221 85 Lund and the Centre for Clinical Research, Uppsala University, Central Hospital, SE 721 89 Västerås, Sweden.

GH is a MD PhD at the Department of Orthopaedics, Lund University, University Hospital, SE 221 85 Lund, Sweden.

## Pre-publication history

The pre-publication history for this paper can be accessed here:

http://www.biomedcentral.com/1471-2431/10/59/prepub
